# Reply to ‘C–C bond cleavage in biosynthesis of 4-alkyl-l-proline precursors of lincomycin and anthramycin cannot precede *C*-methylation’

**DOI:** 10.1038/s41467-018-05500-1

**Published:** 2018-08-09

**Authors:** Guannan Zhong, Hua Chen, Wen Liu

**Affiliations:** 1 State Key Laboratory of Bioorganic and Natural Products Chemistry, Center for Excellence in Molecular Synthesis, Shanghai Institute of Organic Chemistry, Chinese Academy of Sciences, 345 Lingling Road, Shanghai, 200032 China; 2 Huzhou Center of Bio-Synthetic Innovation, 1366 Hongfeng Road, Huzhou, 313000 China

The correspondence by Janata et al.^1^ questions the timing of the activity of LmbA and Ant6, both of which are homologous to γ-glutamyltranspeptidases (γ-GTs) in sequence but do not possess γ-glutamyl transfer/hydrolysis activity that is characteristic for γ-GTs^2,3^, and the methyltransferase activity of LmbW in the formation of a 4-alkyl-l-(dehydro)proline (ALDP) residue shared by lincomycin A and anthramycin. This correspondence essentially is unrelated to our original article^2^, which focuses on the unusual N-terminal nucleophile (Ntn)-hydrolase activity of LmbA/Ant6 and LmbA2991 for a C–C bond cleavage in Gram-positive actinobacteria and does not involve the characterization of LmbW activity and the timing of related reactions during ALDP formation as well. Instead, it is closely related to an earlier work by Janata et al.^4^, in which the authors predicted the function of LmbA but incorrectly assigned this protein as a γ-GT involving a reversible γ-glutamyl transfer activity in C–C bond cleavage and preliminarily assayed LmbW activity in the methylation of intermediate **2/3**, a pyrroline mixture in both imine and enamine forms (Fig. 1a). As a reply to this correspondence, we present evidence herein that C–C bond cleavage does precede the *C*-methylation step in a main ALDP biosynthetic pathway.

**Fig. 1:**
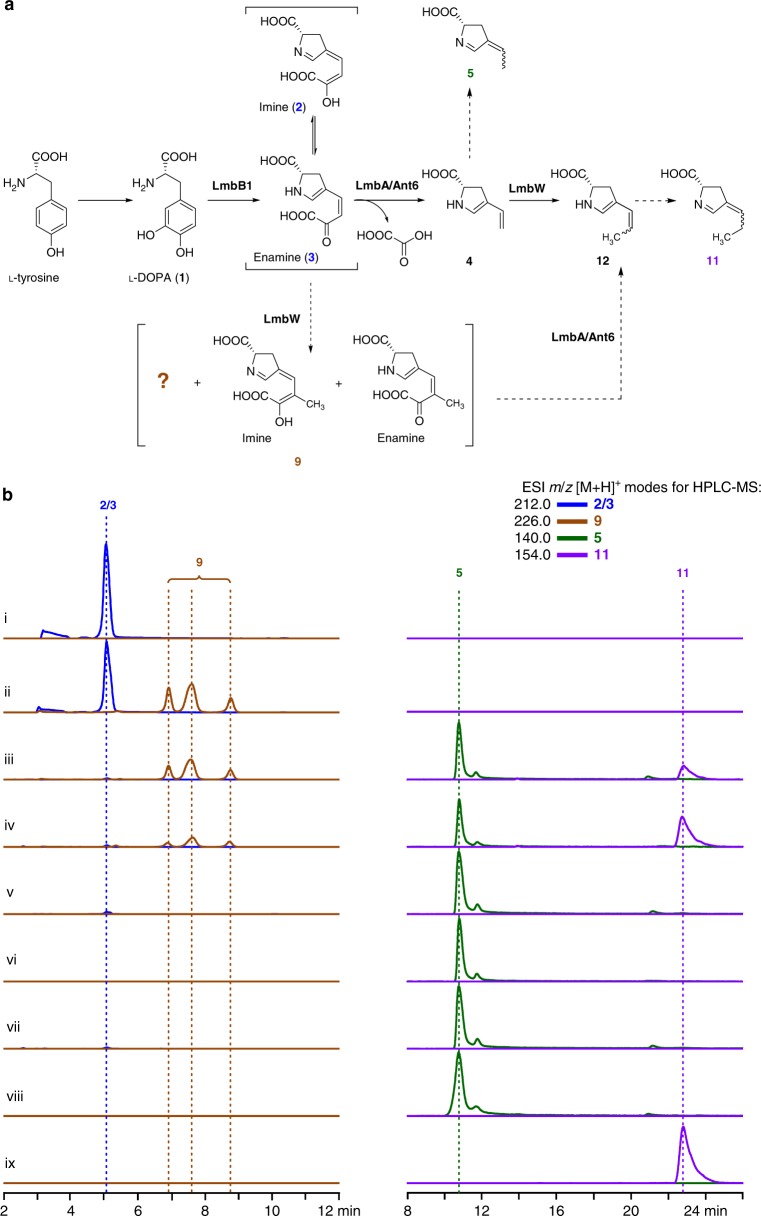
Timing of C–C bond cleavage and *C*-methylation in the formation of ALDP residues. **a** A common pathway for ALDP formation in the biosynthesis of lincomycin A and anthramycin. Main and shunt reactions are indicated by solid and dashed arrows, respectively. **b** Assays of the Ntn-hydrolase activity of Ant6 and the *C*-methyltransferase activity of LmbW by HPLC-MS. Substrate **2**/**3** was prepared in situ through LmbB1-mediated transformation of l-DOPA. i **2/3**; ii incubating **2/3** with LmbW for 1.5 h; iii adding Ant6 into the filtered reaction mixture ii for a further 1.5 h incubation; iv incubating **2/3** with LmbW and Ant6 for 1.5 h; v incubating **2/3** with Ant6 for 1.5 h; vi incubating synthetic **5** with LmbW for 1.5 h; vii adding LmbW into the reaction mixture v for a further 1.5 h incubation; viii standard **5**; and ix standard **11**

According to a procedure described previously^2^, we prepared the unstable pyrroline substrate **2/3** ([M + H]^+^ *m/z*: calcd. 212.0554 for [C_9_H_10_NO_5_]^+^, found 212.0558) in situ using LmbB1-catalyzed dioxygenation reaction to transform l-3,4-dihydroxy-phenylalanine (l-DOPA, **1**). In the presence of LmbW, only a part of **2/3** was converted into a methylated mixture, **9** ([M + H]^+^ *m/z*: calcd. 226.0710 for [C_10_H_12_NO_5_]^+^, found 226.0715), which likely includes both an imine and an enamine and an additional unpredicted product that share a same molecular weight, with a transformation efficiency of ~30.0% over a 1.5 h reaction period (Fig. 1b, ii). The inefficiency of LmbW in transforming **2/3** was also reported in the correspondence by Janata et al.^1^, suggesting that **2/3** is not a favored substrate of LmbW. After filtering this reaction mixture to remove the both catalysts LmbB1 and LmbW, we examined the Ntn-hydrolase activity of the γ-GT homolog Ant6 for C–C bond cleavage in the presence of the both substrates, i.e., unmethylated pyrroline **2/3** (~70.0%) and methylated mixture **9** (~30.0%) (Fig. 1b, iii). Remarkably, **2/3** was converted nearly completely into a diene shunt product **5** (through the tautomerization of **4**) over a 1.5 h period; in contrast, the conversion of **9** proceeded poorly, only yielding a trace of **11** (a methylated **5** derivative, [M + H]^+^ *m/z*: calcd. 154.0863 for [C_8_H_12_NO_2_]^+^, found 154.0863). The identity of **11** to an imine, which likely arises from the tautomerization of the enamine intermediate **12** (Fig. 1a), was confirmed using an authentic compound (Supplementary Figures 2–4 and Supplementary Table 3). These results strongly supported that Ant6 favors unmethylated **2/3** much over methylated **9** as a substrate under same reaction conditions during the catalysis of a C–C bond cleavage (Fig. 1a).

We then validated that the *C*-methylation step can follow C–C bond cleavage in the ALDP biosynthetic pathway. Compared with the above assay that sequentially incorporated LmbW and Ant6, adding the both catalysts simultaneously into the reaction mixture led to a much less accumulation of **9** and a much more production of **11** (~2.1-fold) over a 1.5 h reaction period (Fig. 1b, iv), supporting that LmbW favors the methylation of **4**, the diene product immediately resulting from the Ant6-mediated hydrolysis of **2/3**, instead of this pyrroline intermediate directly during the production of **11** (Fig. 1a). As we reported in the original article^2^, the enamine diene **4** is extremely unstable; and without further enzymatic conversion, it tends to rapidly undergo a tautomerization to the imine **5**, which appears to be resistant to the methylation activity of LmbW and thereby remained in the reaction mixture. The tautomerization of **12** to **11** could share a similar tendency. Indeed, **5** was observed exclusively in the Ant6-catalyzed transformation of **2/3** using a chemically synthesized standard, and LmbW failed to convert **5**, either chemically synthesized or enzymatically prepared (Fig. 1b, v–vii). Based on these experimental data, it is unnecessary to discuss the bioinformatics analysis-based computational study conducted in the correspondence by Janata et al.^1^, which is speculative and attempted to convince that LmbW prefers **2/3** over **4** in the absence of the sufficient biochemical assays and crystal structure of the enzyme.

The correspondence by Janata et al^1^. is misleading in the chemical synthesis of the imine diene product **5**^2^, which was used to prepare an authentic standard for the assay of the Ant6-catalyzed hydrolysis of **2/3** and the examination of the tautomerization tendency of the immediately produced enamine diene **4** by deprotecting **4**′ (a stable enamine mimic), as we described previously^2^. We appreciate a careful reading by Janata et al.^1^, and in Page 39 of Supporting Information associated with our original article^2^, the chemical name of **5**, which incorrectly corresponds to the structure of **4**, should be corrected.

In conclusion, the claims in the correspondence by Janata et al^1^. are incorrect, because they mistakenly assume the substrate preference of both the γ-GT homolog LmbA/Ant6 and the methyltransfearse LmbW based only on a preliminary assay of the methyltransferase activity of LmbW using substrate **2/3** (Janata et al. have never assayed LmbA/Ant6 activity using either **2/3** or **9/10** and LmbW activity using **4**) and a speculative bioinformatics analysis. Taking advantage of responding to this correspondence, we here provide insights into a main ALDP pathway shared by the biosynthesis of lincomycin A and anthramycin, as experimental evidence supports that following the action of an oxidase pair to prepare pyrroline intermediate **2/3**, LmbA/ant6 catalyzes a C–C bond cleavage to yield the unstable enamine diene **4**, which is then *C*-methylated by LmbW for further transformations (Fig. 1a).

**Data availability.** The data are available from the corresponding author on request.

## Electronic supplementary material

Supplementary Information
