# Supplementary material for: Reply to ‘C–C bond cleavage in biosynthesis of 4-alkyl-l-proline precursors of lincomycin and anthramycin cannot precede C-methylation’
Source: Nat Commun. 2018 Aug 9;9:3168. doi: 10.1038/s41467-018-05500-1 (PMC6085292; doi:10.1038/s41467-018-05500-1)
Supplement: Supplementary file 1 — Supplementary Information [file 41467_2018_5500_MOESM1_ESM.pdf]

Supplementary Information

# Correspondence reply: C-C bond cleavage in biosynthesis of 4-alkyl-L-proline precursors of lincomycin and anthramycin cannot precede C-methylation

Guannan Zhong[1], Hua Chen[1] & Wen Liu[1,2,*]

[1] State Key Laboratory of Bioorganic and Natural Products Chemistry, Center for Excellence in Molecular Synthesis, Shanghai Institute of Organic Chemistry, Chinese Academy of Sciences, 345 Lingling Road, Shanghai 200032, China.

[2] Huzhou Center of Bio-Synthetic Innovation, 1366 Hongfeng Road, Huzhou 313000, China.

* To whom correspondence should be addressed: Shanghai Institute of Organic Chemistry, Chinese Academy of Sciences, 345 Lingling Road, Shanghai 200032, China. Wen Liu, Email: wliu@mail.sioc.ac.cn, Tel.: 86-21-54925111, Fax: 86-21-64166128.

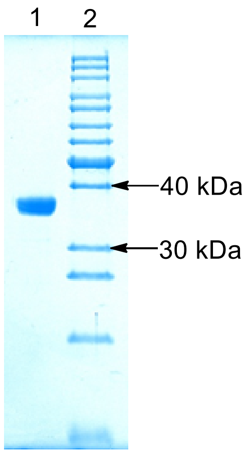

Supplementary Figure 1. Denaturing SDS-PAGE analysis (conc. 10%) of LmbW. Lane 1, LmbW; Lane 2, protein standard.

National Center for Organic Mass Spectrometry in Shanghai
Shanghai Institute of Organic Chemistry
Chinese Academic of Sciences
High Resolution MS DATA REPORT

Instrument: Thermo Fisher Scientific LTQ FT Ultra

Card Serial Number : D151918

Sample Serial Number: 2011117-PPG153

Operator : DONG Date: 2015/07/06

Operation Mode: DART Postive

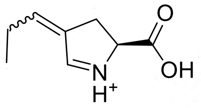

$[M+H]^+$
Chemical Formula: $C_8H_{12}NO_2^+$
Exact Mass: 154.08626
m/z: 154.08680 (100.0%)

Elemental composition search on mass 154.09

m/z= 149.09-159.09

m/z	Theo. Mass	Delta (ppm)	RDB equiv.	Composition
154.0863	154.0863	0.16	3.5	$C_8 H_{12} O_2 N$
	154.0861	1.46	0.0	$C_3 H_{11} O_2 N_4 F$

Supplementary Figure 2. The direct analysis in real time high resolution mass spectrometry (DART-HR-MS) result of compound **11**.

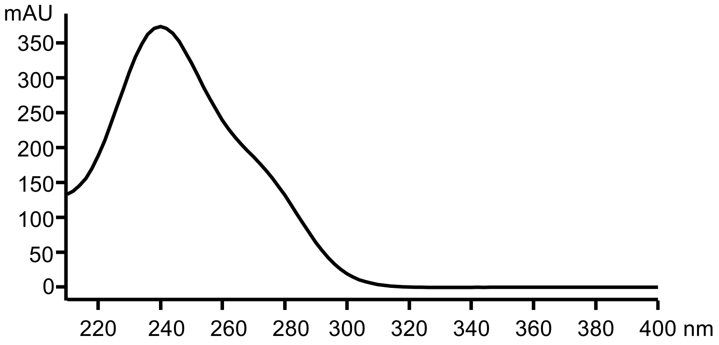

Supplementary Figure 3. Ultraviolet (UV) spectrum of **11** with the maximal absorption at 240 nm.

a: ^{1}H-NMR spectrum (500 MHz, D_2O).

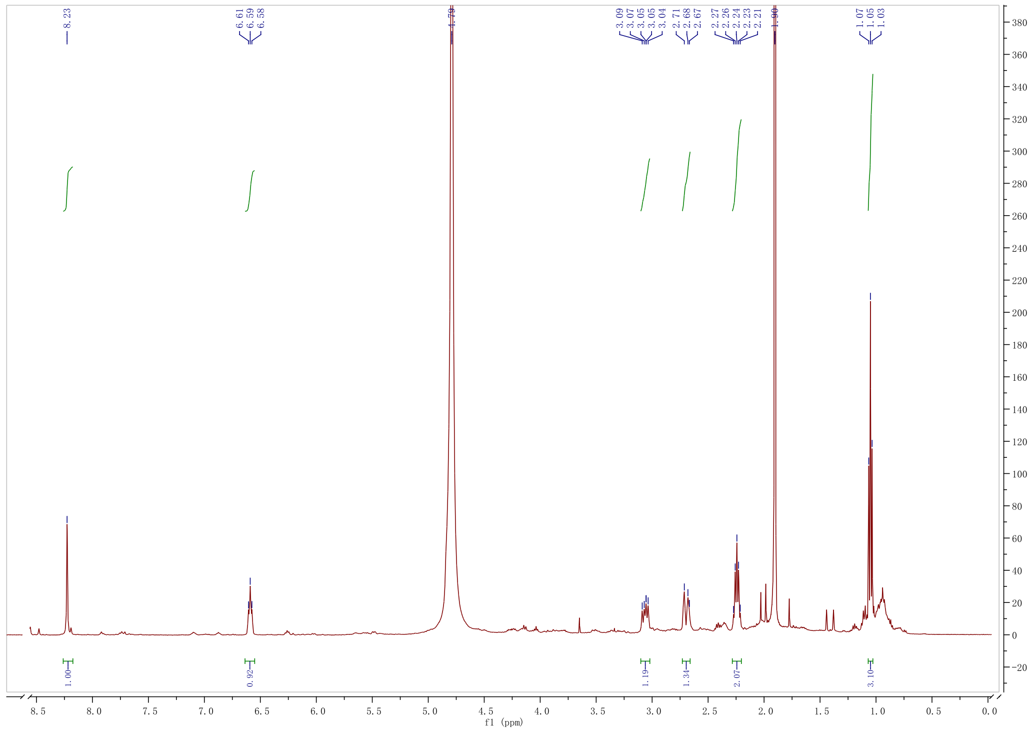

b: ^{13}C-NMR spectrum (125 MHz, D_2O).

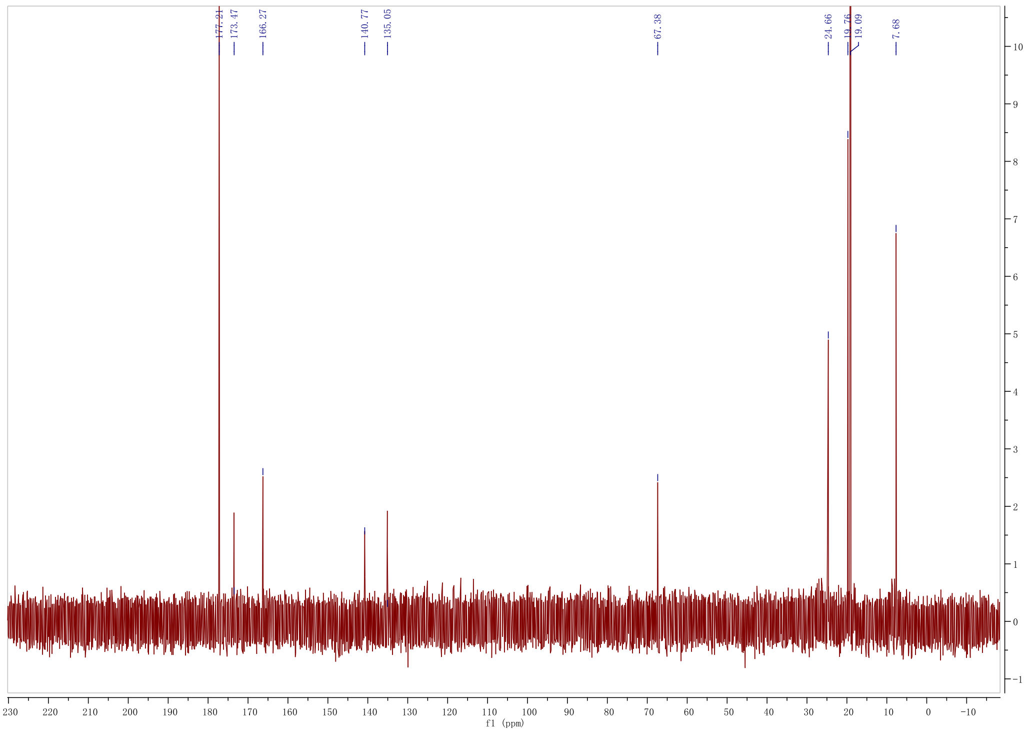

c: H-H COSY spectrum (500 MHz, D_2O).

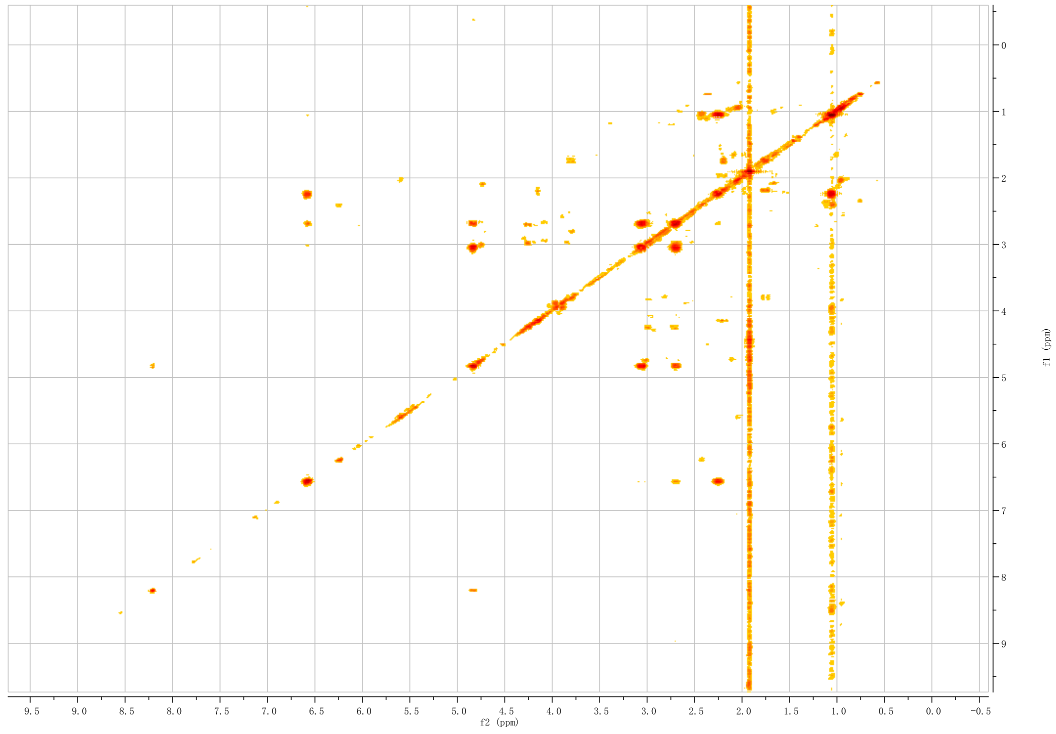

d: H-C HSQC spectrum (D_2O).

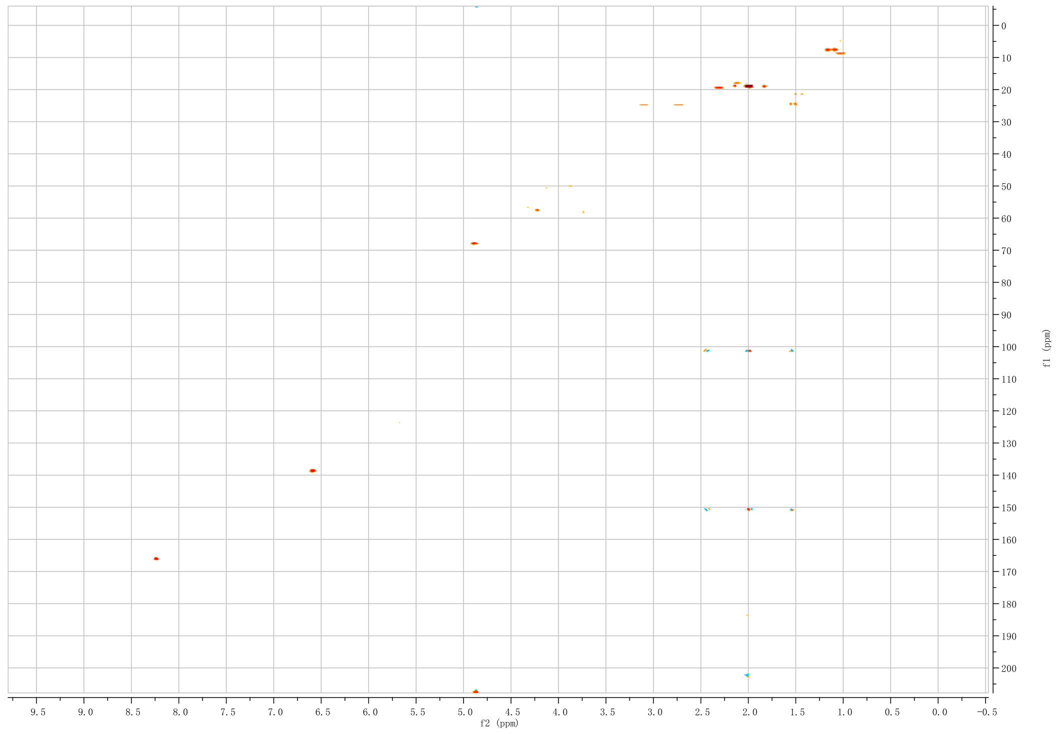

e: H-H NOESY spectrum (500 MHz, D_2O).

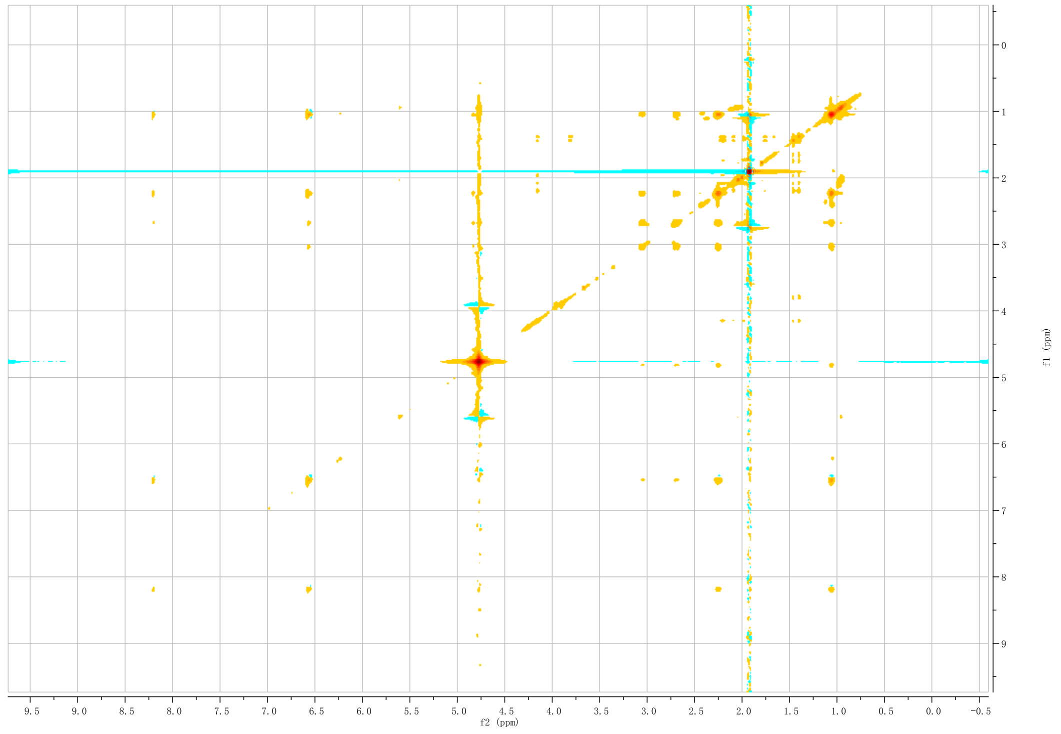

Supplementary Figure 4. NMR spectra of compound **11**. δ/ppm=1.90 (s) in ^{1}H-NMR and δ/ppm=19.09, 177.21 in ^{13}C-NMR are signals for NH_4OAc which could not be removed completely.

a: ^{1}H-NMR spectrum (500 MHz, $CDCl_3$).

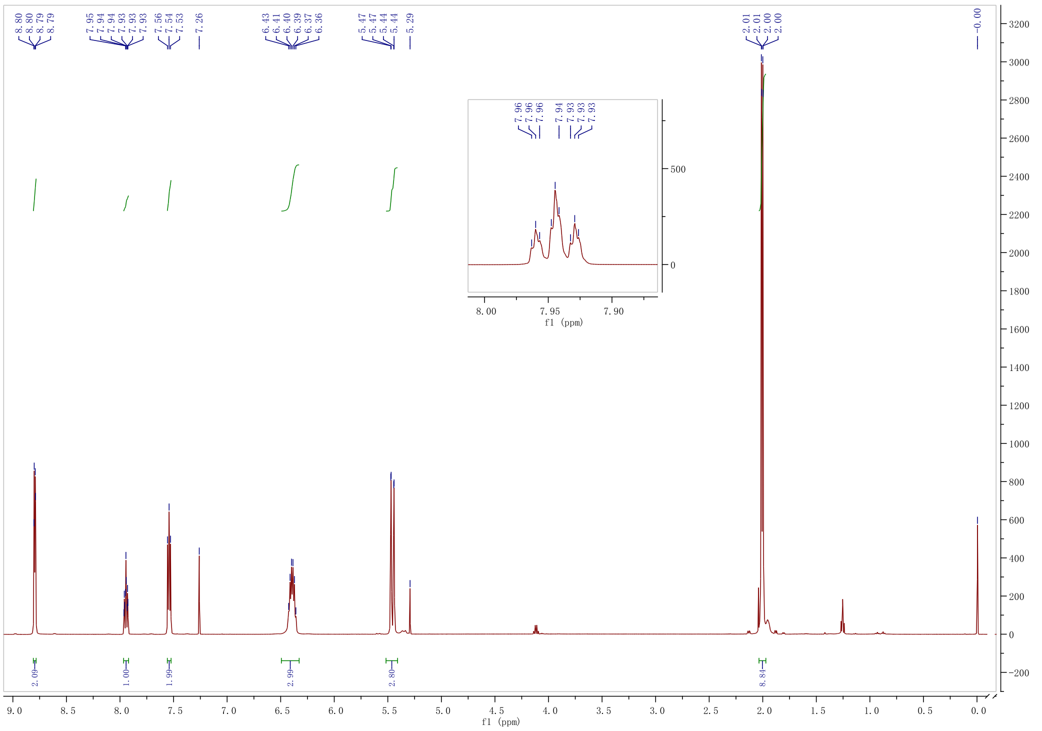

b: ^{13}C-NMR spectrum (125 MHz, $CDCl_3$).

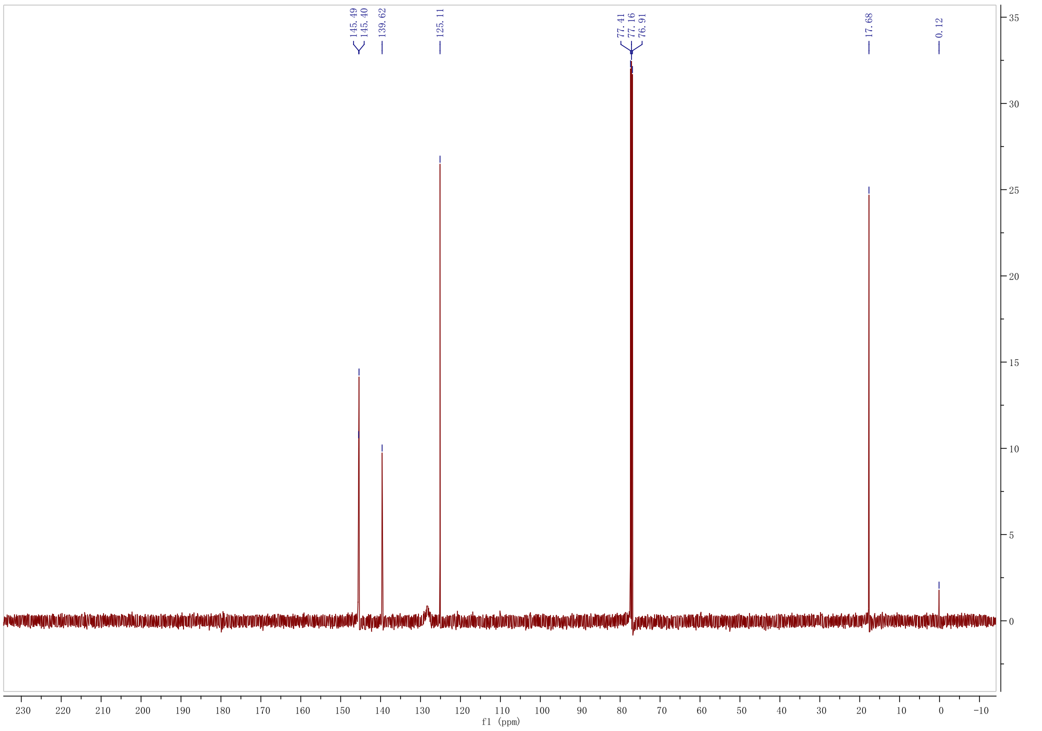

Supplementary Figure 5. NMR spectra of compound **S9**.

a: ^{1}H-NMR spectrum (500 MHz, $CDCl_3$).

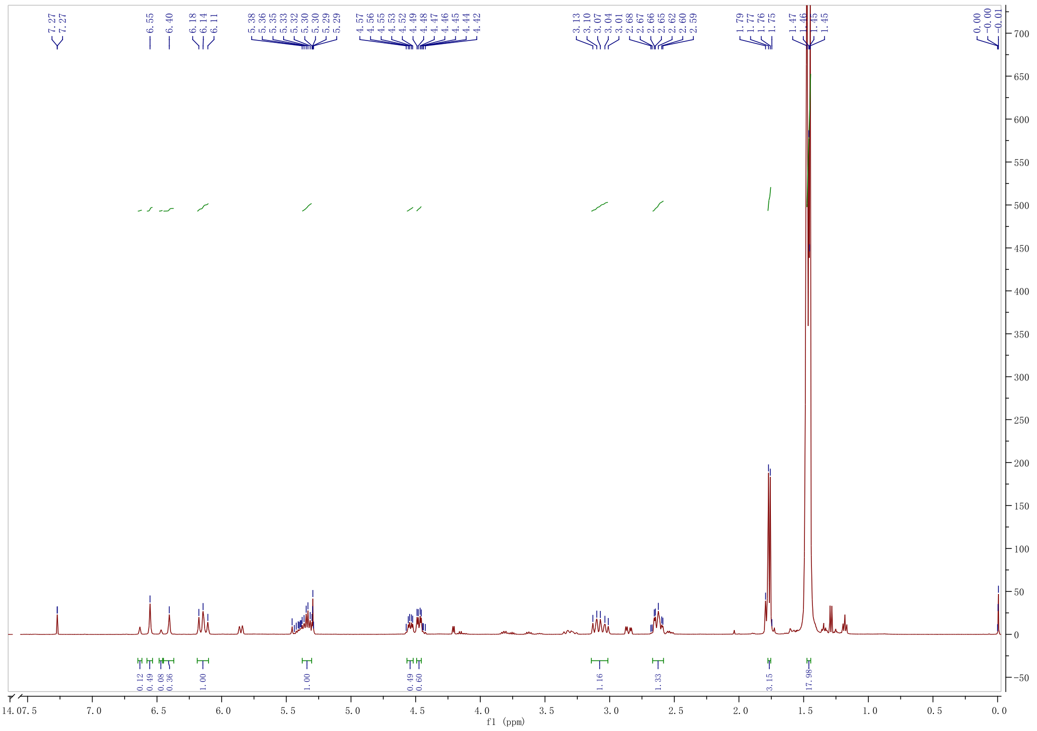

b: ^{13}C-NMR spectrum (125 MHz, $CDCl_3$).

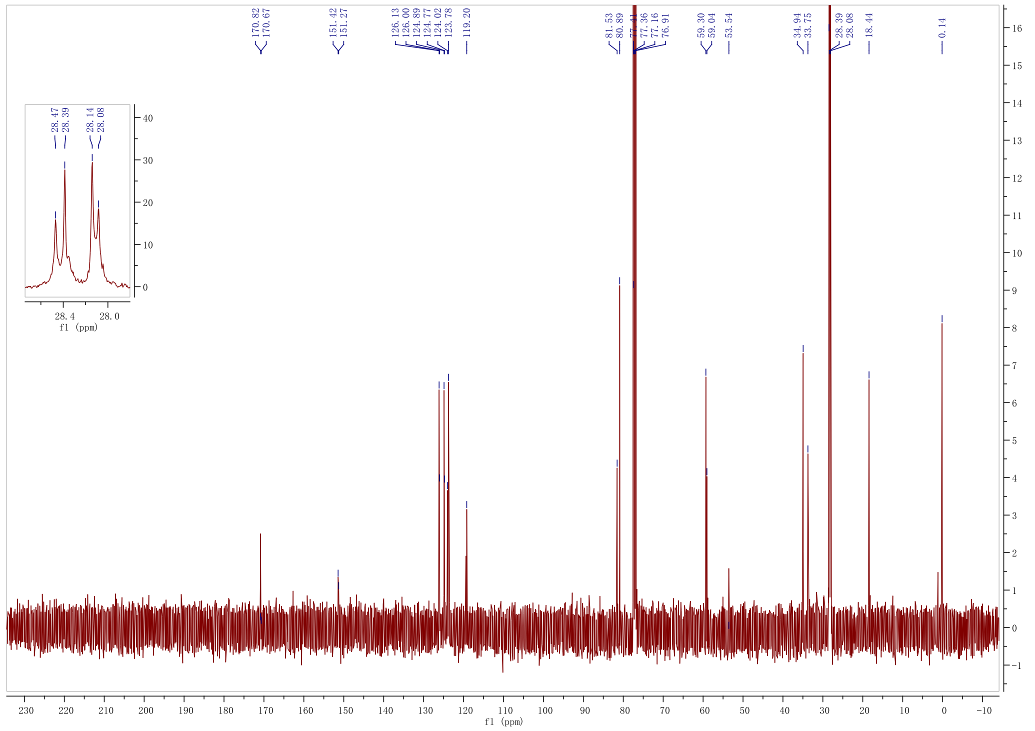

Supplementary Figure 6. NMR spectra of compound **S10**.

Supplementary Table 1. Bacterial strains and plasmids.

Strain/Plasmid	Characteristic(s)	Source/ Reference
S. lincolnensis		
NRRL ISP-5355	Wild type strain, lincomycin-producing, identical to the ATCC 25466 strain	NRRL
E. coli		
DH5α	Host for general cloning	Invitrogen
BL21 (DE3)	Host for protein expression	NEB
LL2009	BL21 (ED3) derivative, containing pLL2012 for producing LmbB1	1
LL2010	BL21 (ED3) derivative, containing pLL2021 for producing Ant6	1
LL2027	BL21 (ED3) derivative, containing pLL2050 for producing LmbW	This study
**Plasmids**		
pMD19-T	*E. coli* subcloning vector	Takara
pET-28a(+)	Protein expression vector used in *E. coli*, encoding *N*-terminal 6× His-tag, kanamycin resistance	Novagen
pACYCDuet-1	Protein co-expression vector used in *E. coli*, encoding *N*- terminal 6×His tag on the former part, chloramphenicol resistance	Novagen
pLL2012	pET28a(+) derivative containing *lmbB1*	1
pLL2021	pACYCDuet-1 derivative containing *ant6* and *ant12*	1
pLL2049	pMD-19T derivative containing *lmbW*	This study
pLL2050	pET28a(+) derivative containing *lmbW*	This study

Supplementary Table 2. Primers used in this study.

Primer	Sequence (5'-3')	
lmbW-for	TATGAATTCCATATGACAGCCGTTCGGCAAAGCC	NdeI
lmbW-rev	TATAAGCTTACTCGAGCCGCCGCGGCACCAGGTCGTAG	HindIII

Supplementary Table 3. NMR spectroscopic data for compound **11**. In D_2O, 500 MHz for ^1H and 125 MHz for ^{13}C NMR. Chemical shifts are reported in ppm.

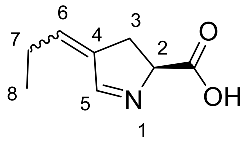

Position	mutl.	δ_C	δ_H (mult., *J* in Hz)
2	CH	67.38	4.84
2-C=O	C=O	173.47	-
3a	CH_2	24.66	2.68 (1H, d, 16.5)
3b	CH_2	24.66	3.05 (1H, dd, 17.5, 9)
4	C_{quart}	140.77	-
5	CH	166.27	8.23 (1H, s)
6	CH	135.05	6.59 (1H, t, 7.5)
7	CH_2	19.76	2.24 (2H, qd, 7.5, 7.5)
8	CH_3	7.68	1.05 (3H, t, 7.5)

Supplementary Methods

Materials and methods

Materials, bacterial strains and plasmids. Biochemicals and media were purchased from Sinopharm Chemical Reagent Co. Ltd. (China) or Oxoid Ltd. (UK) unless stated. Enzymes were purchased from Takara Biotechnology Co. Ltd. (China) except Taq DNA polymerase from Dingguo Co. Ltd. (China). Chemical compounds and reagents were purchased from Sigma-Aldrich Co. (USA), TCI Development Co. Ltd. (Japan) and J&K Scientific Ltd. (China). Tetrahydrofuran (THF) and toluene were distilled from sodium prior to use. Dichloromethane and pyridine were distilled from calcium hydride. All reactions involving moisture sensitive reactants were executed under argon atmosphere using oven dried and/or flame dried glassware. All other solvents, reagents and chemicals were used as purchased unless stated otherwise. Bacteria strains and plasmids used in this study are listed in Supplementary Table 1. Primers used in this study are summarized in Supplementary Table 2.

Analysis. High performance liquid chromatography (HPLC) analysis was carried out on Agilent 1200 HPLC system (Agilent Technologies Inc., USA). Electrospray ionization mass spectrometry (ESI-MS) was performed on a Thermo Fisher LTQ XL ESI-MS spectrometer (Thermo Fisher Scientific Inc., USA), and the data were analyzed using Thermo Xcalibur software. ESI-high resolution MS (ESI-HR-MS) analysis was carried out on 6230B Accurate-Mass TOF LC/MS System or 6530 Accurate-Mass Q-TOF LC/MS System (Agilent Technologies Inc., USA) and the

data were analyzed using Agilent MassHunter Qualitative Analysis software. Direct analysis in real time high resolution mass spectrometry (DART-HR-MS) analysis was carried out on a Thermo Fisher Scientific LTQ FT Ultra spectrometer (Thermo Fisher Scientific Inc., USA). Electron impact mass spectrometry (EI-MS) was performed on Agilent Technologies 5793N (Agilent Technologies Inc., USA). EI-HR-MS was carried out on Waters Micromass GCT Premier (Waters Corporation, USA). NMR data were recorded on the Bruker AV500 spectrometers (Bruker Co. Ltd, Germany) or on the Agilent 500 MHz Premium Compact+ NMR spectrometer (Agilent Technologies Inc., USA).

Protein expression and purification

LmbB1 and Ant6. LmbB1 and Ant6 were expressed and purified according to the previously described method[1].

LmbW. A 1050 bp DNA fragment containing *lmbW* was amplified by PCR using the primers lmbW-for and lmbW-rev and then cloned into pMD19-T to yield pLL2049. After sequencing to validate the fidelity, the *lmbW* fragment recovered from pLL2049 was inserted into the *Nde*I-*Hind*III site of pET28a(+) to yield the recombinant plasmid pLL2050. The resulting plasmid pLL2050 was transferred into *E. coli* BL21 (DE3) for expression. LmbW, fused to an *N*-terminal 6 x His tag was expressed at 25 ℃ for 24 h with 100-μM isopropyl-β-D-thiogalactopyranoside (IPTG, added at OD_{600} = 0.6) induction and shaking at 220 rpm. Cells were harvested by centrifugation at 4 ℃ and re-suspended in lysis buffer containing 50-mM K_2HPO_4 (pH 8.0), 300-mM NaCl, 5-mM imidazole and 10% (v/v) glycerol. After disruption by a low-temperature ultra-high-pressure cell disrupter, the

insoluble material was removed by centrifugation at 15000 g at 4 ℃. The soluble fraction was subjected to purification using a HisTrap FF column (GE Healthcare, USA) according to the manufacturer's protocol. The elution fraction containing the recombinant protein was desalted using a PD-10 Desalting Column (GE Healthcare, USA) into storage buffer (50-mM K_2HPO_4 (pH 8.0), 100-mM NaCl, 10% (v/v) glycerol and 1-mM DTT). The resulting protein was concentrated and stored at -80 ℃. The purity of the protein was determined by 10% sodium dodecyl sulfate polyacrylamide gel electrophoresis (SDS-PAGE) analysis, and the concentration was determined by the Bradford assay using bovine serum albumin (BSA) as the standard.

***In vitro* enzymatic assay**

Each of the assays was performed at 30 ℃ in 100-mM K_2HPO_4 buffer (pH 8.0) with a total volume of 100 μL. The original reaction mixture contained approximately 500-μM L-DOPA, 2.5-mM $MgCl_2$ and 2.5-mM *S*-adenosyl-L-methionine (SAM).

For the preparation of **2/3**, 10-μM LmbB1 was added to the original reaction mixture for a 1.5 h incubation. For assaying methyltransferase activity or Ntn-hydrolase activity alone, 10-μM LmbW or 10-μM Ant6 was added with 10-μM LmbB1 for a 1.5 h incubation. For assaying methyltransferase activity and Ntn-hydrolase activity in tandem, 10-μM LmbW was added with 10-μM LmbB1 for a 1.5 h incubation, and then Microcon-10 kDa Centrifugal Filter Unit with Ultracel-10 membrane (Merck Millipore Ltd., USA) was used for centrifugation (25 min at 13,000 g) to remove LmbB1 and LmbW before 10-μM Ant6 was added into the filtered reaction mixture for a

further 1.5 h incubation. For assaying methyltransferase activity and Ntn-hydrolase activity simultaneously, 10-μM LmbW and 10-μM Ant6 were added with 10-μM LmbB1 for a 1.5 h incubation. For assaying Ntn-hydrolase activity and methyltransferase activity in tandem, 10-μM Ant6 was added with 10-μM LmbB1 for a 1.5 h incubation before 10-μM LmbW was added for a further 1.5 h incubation. Assay the methylation of **5**, 10-μM LmbW was added to the reaction mixture containing 400-μM compound **5**, 2.5-mM $MgCl_2$ and 2.5-mM SAM for a 1.5 h incubation..

For the examination of pyrroline intermediates **2/3** and **9**, the assays were quenched by adding a 1% volume of HCOOH. For the examination of imine dienes **5** and **11**, the assays were quenched by adding an equal volume of CH_3OH. After centrifugation (5 min at 13,000 g), the supernatant was analyzed by HPLC-ESI-MS using a Phenomenex column (Luna 5 μ C18(2) 100A, 4.60 x 250 mm, 5 micron, Phenomenex Inc., USA) with a gradient elution of solvent A (H_2O containing 10 mM NH_4OAc) and solvent B (CH_3OH) at a flow rate of 1 mL min^{-1} and ultraviolet (UV) detection at 240 nm using a 30-min gradient program: t = 0 min, 5% B; t = 5 min, 5% B; t = 15 min, 15% B; t = 25 min, 15% B; t = 26min, 5% B and t = 30 min, 5% B.

Chemical synthesis

Compound **11** was synthesized according to the methods previously described[1].

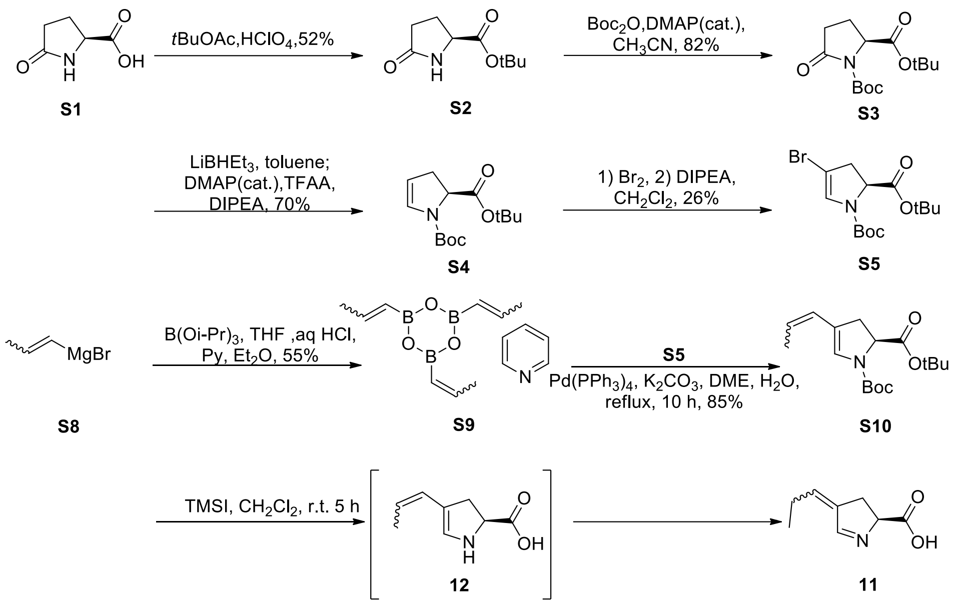

2,4,6-tri(prop-1-en-1-yl)-1,3,5,2,4,6-trioxatriborinane compound with pyridine (S9). Yellow solid. **^{1}H-NMR** (500 MHz, $CDCl_3$): δ/ppm=2.00 (dd, *J*=8, *J*=1 Hz, 9H), 5.46 (dd, *J*=13.5, *J*=1.5 Hz, 3H), 6.36-6.43 (m, 3H), 7.54 (t, *J*=7 Hz, 2H), 7.93-7.95 (m, 1H), 8.79-8.80 (m, 2H). **^{13}C-NMR** (125 MHz, $CDCl_3$): δ/ppm=17.68, 125.11, 139.62, 145.40, 145.49. **MS** (70 eV, EI), m/z (%): 204 (0.31) [$M^+–C_5H_5N$], 79 (100) [$C_5H_5N^+$]. **EI-HRMS** ($C_9H_{15}O_3{}^{10}B_3$): calcd. 201.1409; found 201.1411.

(*S*)-di-*tert*-butyl 4-(prop-1-en-1-yl)-2,3-dihydro-1*H*-pyrrole-1,2-dicarboxylate (S10). Yellow oil. **^{1}H-NMR** (500 MHz, $CDCl_3$): δ/ppm=1.45, 1.46, 1.47 (3×s, 18H), 1.76 (d, *J*=7 Hz, 3H), 2.59-2.66 (td, *J*=12.5 Hz, *J*=4.5 Hz, 1H), 3.01-3.13 (m, 1H), 4.42-4.49 (dd, *J*=12 Hz, *J*=5 Hz, 0.5H), 4.52-4.57 (dd, *J*=12 Hz, *J*=5 Hz, 0.5H), 5.29-5.45 (m, 1H), 6.14 (t, *J*=18.5 Hz, 1H), 6.40 (s, 0.5H), 6.55 (s, 0.5H). **^{13}C-NMR** (125 MHz, $CDCl_3$): δ/ppm=18.43 (+), 28.08 (+), 28.14 (+), 28.38 (+), 28.46 (+), 34.94 (-), 59.04 (+), 59.30 (+), 80.89 (C_{quart}), 81.54 (C_{quart}), 119.21 (+), 122.82 (+), 123.78 (+), 124.88 (+), 126.12 (+), 128.92 (+), 151.46 (C_{quart}), 170.86 (C_{quart}). **MS** (70 eV, EI), m/z (%):

309 (13.86) [M^+], 209 (17.49) [M^+–$CO_2C(CH_2)(CH_3)_2$], 153 (100) [M^+–$CO_2C(CH_2)(CH_3)_2$–$C(CH_2)(CH_3)_2$], 108 (48.24) [$C_7H_{10}N^+$], 57 (32.03) [$C_4H_9^+$]. **EI-HRMS** ($C_{17}H_{27}NO_4$)： calcd. 309.1940, found 309.1937.

(*S*)-4-propylidene-3,4-dihydro-2*H*-pyrrole-2-carboxylic acid (11). Yellow oil. **^{1}H-NMR** (500 MHz, D_2O): δ/ppm=1.05 (t, *J*=7.5 Hz, 3H), 2.24 (qd, *J*=7.5 Hz, *J*=7.5 Hz, 2H), 2.68 (d, *J*=16.5 Hz, 1H), 3.05 (dd, *J*=17.5 Hz, *J*=9 Hz, 1H), 6.59 (t, *J*=7.5 Hz, 1H), 8.23 (s, 1H). **^{13}C-NMR** (125 MHz, D_2O): δ/ppm=7.68 (+), 19.76 (-), 24.66 (-), 67.38 (+), 135.05 (+), 140.77 (C_{quart}), 166.27 (+), 173.47 (C_{quart}). DART-HR-MS [$C_8H_{12}NO_2$]$^+$: calcd. 154.0863, found 154.0863.

Supplementary References

1. Zhong, G., Zhao, Q., Zhang, Q. & Liu, W. 4-alkyl-L-(Dehydro)proline biosynthesis in actinobacteria involves *N*-terminal nucleophile-hydrolase activity of γ-glutamyltranspeptidase homolog for C-C bond cleavage. *Nat. Commun.* **8**, 16109 (2017).
